# HIV risks and vulnerabilities reported among adolescent girls and young women accessing DREAMS services in three Zambian districts: Monze, Mazabuka, and Mongu, 2020–2022

**DOI:** 10.1186/s12981-026-00848-7

**Published:** 2026-02-10

**Authors:** Kalima Tembo, Caitlin Baumhart, Linah Mwango, Brianna Lindsay, Pawel Olowski, Julian Chipukuma, Adebayo Olufunso, Morley Mujansi, Boyd Kaliki, Omega Chituwo, Carlos Muleya, Annie Mwila, Cassidy W. Claassen

**Affiliations:** 1 Maryland Global Initiatives Corporation Zambia, 31C Bishops Rd, Lusaka, Zambia; 2 Center for International Health, Education, and Biosecurity, University of Maryland School of Medicine, Baltimore, MD USA; 3 Ciheb Zambia, Lusaka, Zambia; 4 Division of Global HIV and TB, U.S. Centers for Disease Control and Prevention, Lusaka, Zambia; 5 UNICEF Zambia, Lusaka, Zambia; 6 Institute of Human Virology, University of Maryland School of Medicine, Baltimore, MD USA

**Keywords:** DREAMS, HIV risks and vulnerabilities, Adolescent girls and young women, HIV prevention, Zambia, Sub-Saharan Africa

## Abstract

**Background:**

Adolescent girls and young women (AGYW) in sub-Saharan Africa face socio-economic and gender-related factors that heighten risk of HIV infection. We examined HIV risks and vulnerabilities among AGYW enrolled in the Determined Resilient Empowered AIDS-free Mentored and Safe (DREAMS) program in Zambia.

**Methods:**

AGYW aged 10–24 years were screened at enrollment in DREAMS using a standardized tool assessing exposure to sexual and reproductive health risks, gender-based violence, and orphanhood. We analyzed 24 months of data (October 2020–September 2022) from six centers in Southern and Western Provinces. We characterized age-disaggregated risks and used multivariable logistic regression to examine associations with engagement in HIV-related clinical services.

**Results:**

Among 63,118 AGYW screened, 34.9% were aged 10–14 years, 50.7% aged 15–19 years, and 14.4% aged 20–24 years. Emotional or physical violence (78.0%) and orphanhood (22.3%) were most common among girls aged 10–14 years. Among AGYW 15–19 years, top risks included no/irregular condom use (58.1%), orphanhood (29.2%), multiple partners (23.5%), and transactional sex (20.1%). In the 20–24 years group, 83.5% reported no/irregular condom use, with 29.9% reporting multiple partners and 23.0% transactional sex. Clinical service engagement was positively associated with being out of school, sexually active, using drugs/alcohol, and reporting prior pregnancy, STIs, or multiple sexual partners.

**Conclusion:**

Socio-economic vulnerabilities were common among younger AGYW in Zambia, while older AGYW reported high levels of behavioral HIV risk. DREAMS reached vulnerable and at-risk AGYW in Zambia, representing an opportunity to reduce HIV acquisition through targeted prevention services.

**Supplementary Information:**

The online version contains supplementary material available at 10.1186/s12981-026-00848-7.

## Introduction

Across Africa, adolescent girls and young women (AGYW) are at significantly higher risk for HIV infection than other demographic groups. Over 77% of new HIV infections occur in AGYW aged 15–24 years in sub-Saharan Africa (SSA), with more than 3,100 AGYW acquiring HIV weekly [1]. AGYW are also three times more likely to acquire HIV compared to their adolescent male peers [1].

In addition to inherent biological susceptibility, multiple interlocking biological, socio-economic, and structural factors increase HIV risk among AGYW. Many young women in SSA experience socioeconomic vulnerabilities such as poverty, early sexual debut, food insecurity, unstable housing, limited education access, intimate partner violence, and sexual abuse [2–4]. These factors often lead to HIV-related risks, including transactional and intergenerational sexual relationships seeking financial or social security, complicated by limited access and empowerment to use condoms, family planning, and other sexual reproductive health (SRH) measures [2–4]. By age 19, 53% of Zambian AGYW have had a child or are pregnant [5]; limited access to SRH puts both the young women and their infants at risk for HIV acquisition.

Zambia has a large proportion of adolescents, with youth aged 10–19 years comprising over 25% of the country’s population [5]. In Zambia the overall HIV prevalence for people aged 15 + years is 11.0%, with women (13.9%) being disproportionally affected compared to men (8.0%) [6]. Zambia has almost four million AGYW; HIV prevalence among AGYW aged 15–19 years is 1.9%, rising to 5.9% among AGYW aged 20–24 years [6]. AGYW face greater risks of acquiring HIV than adolescent boys and young men, while only 42.6% of AGYW 15–24 years old have knowledge of HIV prevention [7]. Despite this enhanced risk, few services exist to support AGYW; health services are centered around pregnant women and children under 5. AGYW face challenges in accessing services and support around gender-based violence (GBV), SRH, and HIV prevention due to social and structural barriers such as health misconceptions, parental and provider disapproval, and intersectional stigma [8, 9].

The Determined, Resilient, Empowered, AIDS-free, Mentored, and Safe (DREAMS) project is a PEPFAR-funded initiative to combat structural HIV-related risks and socioeconomic vulnerabilities for AGYW who meet eligibility criteria [10]. DREAMS is a multi-faceted set of interventions that provides SRH services, education support, and economic empowerment to help keep AGYW free from HIV. By establishing physical safe spaces with supportive community environments, DREAMS provides evidence-based interventions to reduce HIV risk and mitigate socioeconomic vulnerabilities, including clinical services such as HIV testing, pre-exposure prophylaxis (PrEP), and family planning.

The Zambian Ministry of Health (MOH) implemented DREAMS in Zambia in 2015 with support from PEPFAR and implementing partners [11]. The University of Maryland Baltimore (UMB) has been implementing DREAMS in Zambia under the Community Impact to Reach Key and Underserved Individuals for Treatment and Support (CIRKUITS) and Zambia Community HIV Epidemic Control for Key Populations (Z-CHECK) projects since 2020.

We sought to characterize prevalence and frequency of established HIV risks and socioeconomic vulnerabilities among DREAMS participants aged 10–24 years in Zambia. Secondary objectives included describing the proportion of AGYW who engaged in clinical services through DREAMS and examining possible associations between reported risk factors and engagement in clinical services.

## Methods

### Study design and population

This was a retrospective cross-sectional study of existing observational screening data to assess risks and vulnerabilities among AGYW enrolled in the DREAMS program. This study was a secondary data analysis that examined AGYW aged 10–24 years enrolled in DREAMS between October 2020 and August 2022 in three districts in Zambia (Mongu, Monze, and Mazabuka). Mongu District is the capital of Western Province with 13.6% provincial HIV prevalence [12]; Monze and Mazabuka Districts are located in Southern Province with a provincial HIV prevalence of 13.2% [12].

### DREAMS program

#### Enrollment

Community mobilization and sensitization sessions on the DREAMS initiative were provided to AGYW and their parents/guardians to provide information about DREAMS and explain benefits of participation. These sessions provided a basis for informed consent for both AGYW and parents/guardians. The consenting and assenting process for enrolment into DREAMS was described prior to service consent form distribution to ensure autonomy and self-determination. For those who desired to participate in DREAMS, AGYW aged 10–18 years signed the minor assent form and parents/guardians signed the consent forms; AGYW aged 19–24 years signed consent forms themselves. The DREAMS enrollment eligibility screening tool was administered to all consenting AGYW.

#### Screening

AGYW were screened for HIV risks (low condom use, transactional sex, multiple partners, etc.) and socioeconomic vulnerabilities (i.e. GBV, orphanhood) using the structured DREAMS HIV risk assessment tool to determine the applicable secondary prevention services (Table 1). The questionnaire was categorized into age groups with each group having questions tailored to their age; six questions for individuals aged 10–14 years, nine questions for individuals aged 15–19 years, and six questions for individuals aged 20–24 years. The questionnaire enlisted risks on emotional or physical abuse or child neglect, alcohol abuse, out of school, orphanhood, engagement in sexual activity, multiple sexual partners, ever pregnant or having a child, sexually transmitted infection (STI) diagnosis, irregular condom use, victim of sexual violence, and transactional sex, including staying in a relationship for material or financial support, including sexual exploitation of minors. AGYW were asked whether they had experienced each risk and to indicate all risks that applied to them. AGYW reporting one or more risks and vulnerabilities were enrolled into DREAMS. All AGYW were offered the Stepping Stones curriculum [13], a primary package of services containing mentorship sessions covering content such SRH, GBV, and financial literacy. Depending on risks and vulnerabilities identified, AGYW were provided with layered services, ranging from educational and vocational support, economic strengthening, a parenting program, and clinical services such as HIV testing and PrEP.

**Table 1:** Demographics and characteristics of eligible AGYW by District, Zambia October 2020 to August 2023

10–14 years
Q1: Ever been pregnant or pregnant or has a child?
Q2: Victim of emotional or physical violence or abuse or neglect
Q3: Alcohol or drug use
Q4: Out of school
Q5: Orphan hood
Q6: Engages in sexual activity
15–19 years
Q1: Had multiple sexual partners in the past one year
Q2: Ever been pregnant or pregnant or hasa child
Q3: Have you ever been diagnosed with an STI
Q4: No or Irregular condom use
Q5: Transactional sex including staying in a relationship for material or financial support
Q6: Experience of sexual violence (Lifetime)
Q7: Is engaged in alcohol or drug abuse
Q8: Out of school
Q9: Orphan hood
20–24 years
Q1: Had multiple sexual partners in the past one year
Q2: Have you ever been diagnosed with an STI
Q3: No or Irregular condom use
Q4: Transactional sex including staying in a relationship for material or financial support
Q5: Experience of sexual violence (Lifetime)
Q6: Is engaged in alcohol or drug abuse

#### Sexually exploited minors (under 18 years)

Pregnant or parenting sexually exploited minors (those under 18 and reported exchanging sex for money or goods) received comprehensive legal, health, and social support, with Zambia’s child protection laws prioritizing rehabilitation. Public health facilities provided medical and psychological care, while social welfare programs offered counseling, legal aid, and efforts to reintegrate minors into schools or vocational training. The extent to which services could be provided were sometimes limited due to persistent challenges like social stigma, limited resources, and coordination issues.

### Inclusion criteria, exposures, and outcomes

**Fig. 1:**
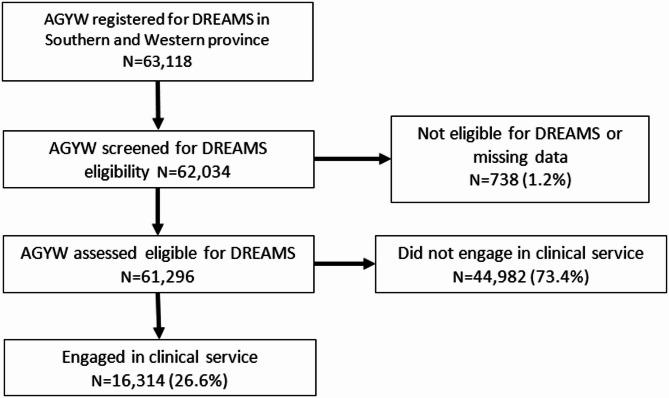
DREAMS adolescent girl and young women registration, eligibility and engagement in clinical service in Mongu, Monze, and Mazabuka Districts in Zambia, Oct 2020 to Aug 2022

For this analysis, the inclusion criteria included: (1) AGYW who were screened; (2) found eligible for and enrolled in DREAMS programs; and (3) in Mazabuka, Monze, and Mongu Districts, Zambia. AGYW with missing data on the DREAMS eligibility screening form were excluded (Fig. 1). Primary outcomes of interest included risks and vulnerabilities reported by AGYW on the DREAMS eligibility screening form. Clinical service engagement was defined as the proportion of AGYW enrolled in DREAMS who engaged in at least one clinical service (i.e., HIV testing, PrEP, family planning, or condoms) between October 2020 and August 2022. Exposure variables used to assess the factors associated with engagement in clinical services included self-reporting of health, behavioral, and socio-behavioral factors on the DREAMS eligibility screening form (supplemental). We disaggregated outcomes data by age groups and districts. To define outcomes and age groups, we used the PEPFAR Monitoring, Evaluation, and Reporting (MER) Indicator Reference Sheet version 2.4 (PEPFAR, n.d.).

### Data sources and statistical analysis

We examined data from AGYW registered for DREAMS in Southern and Western Provinces within the UMB client-level District Health Information Software 2 database (DHIS-2 n.d.). For descriptive analysis, frequency distributions, proportions, and summary statistics were calculated on risk factors and outcomes of interest. Pearson chi-square test was used to test for statistically significant differences in outcomes by categorical variables. Because our dependent variable was bivariate, multivariable logistic regression was used to generate adjusted odds ratios (aOR) and 95% confidence intervals (95%CI) to measure factors associated with engaging in at least one clinical service through the DREAMS program. Separate models were used for each age group. In each model, HIV risks and vulnerabilities were considered as the independent variables and engagement in clinical service as the dependent variable. All analyses were performed using Stata17 SE (STATA Corporation, College Station, TX) and R software-version 4.0.3 (R Core Team 2021).

### Ethical approval

This study was reviewed and approved by the institutional review boards (IRB) at the ERES Converge Zambian IRB (2021-Jan-010, 2020-Mar-015), National Health Research Authority (NHRA0008/15/03/2021, NHRA00029/04/2020), and University of Maryland School of Medicine (HP-00096480, HP-00086064). Associated footnote: See 45 C.F.R. part 46.101(c);21 C.F.R. part 56. Informed consent was waived by the IRBs as this study was a retrospective review of de-identified existing program data.

## Results

### Characteristics of DREAMS AGYW

Of the 63,118 AGYW registered within the data system for DREAMS in Southern and Western Provinces Zambia, 98.3% (62,034/63,118) were screened for eligibility and 97.1% (61,296/63,118) were eligible (Fig. 1). Among eligible AGYW, 34.9% (21,374/61,296) were aged 10–14 years, 50.7% (31,073/61,296) were aged 15–19 years and 14.4% (8,849/61,296) were aged 20–24 years (Table 2). In this sample, 35.6% (21,835/61,296) were assessed in Mazabuka District, Southern Province, 35.3% (21,626/61,296) were assessed in Monze District, Southern Province, and 29.1% (17,835/61,296) were assessed in Mongu District, Western Province. In Mongu, a higher proportion of AGYW were assessed from the 15–19 age group (65.6%) than in Mazabuka (47.6%) and Mongu (41.5%). Mazabuka and Monze had greater representation in the 10-14- and 20-24-years age groups compared to Mongu. The large majority 95.6% (58,583/61,296) of AGYW were single and 4.3% (2,650/61,296) were married; however, marriage was more common in Southern Province districts of Monze and Mazabuka (5.0% and 6.5%) than in Mongu, Western Province (0.8%) (Table 2). Of 61,296 AGYW who were found eligible for DREAMS, 26.6% (16,314/61,296) engaged in clinical service while 73.4% (44,982/61,296) did not (Fig. 1).

**Table 2:** Demographics and characteristics of eligible AGYW by District, Zambia October 2020 to August 2023

	Mongu	Mazabuka	Monze	Total
	(*N* = 17835)	(*N* = 21835)	(*N* = 21626)	(*N* = 61,296)
Age				
Median (Q1-Q3)	15.0 (14.0–17.0)	15.0 (13.0–18.0)	15.0 (12.0–18.0)	15.0 (13.0–18.0)
SD	2.81	3.65	3.87	3.52
	N (%)	N(%)	N (%)	N(%)
Age category				
10–14 years	5227 (29.3%)	7727 (35.4%)	8420 (38.9%)	21,374 (34.9%)
15–19 years	11,704 (65.6%)	10,387 (47.6%)	8982 (41.5%)	31,073 (50.7%)
20–24 years	904 (5.1%)	3721 (17.0%)	4224 (19.5%)	8849 (14.4%)
Birth province				
Southern	35 (0.2%)	20,543 (94.1%)	20,684 (95.6%)	41,262 (67.3%)
Western	17,678 (99.1%)	144 (0.7%)	95 (0.4%)	17,917 (29.2%)
Lusaka	23 (0.1%)	547 (2.5%)	360 (1.7%)	930 (1.5%)
Other	99 (0.6%)	601 (2.8%)	486 (2.2%)	1186 (1.9%)
Missing	0 (0%)	0 (0%)	1 (0.0%)	1 (0.0%)
Marital status				
Single	17,685 (99.2%)	20,700 (94.8%)	20,198 (93.4%)	58,583 (95.6%)
Married	145 (0.8%)	1100 (5.0%)	1405 (6.5%)	2650 (4.3%)
Divorced	3 (0.0%)	29 (0.1%)	21 (0.1%)	53 (0.1%)
Widowed	0 (0%)	5 (0.0%)	0 (0%)	5 (0.0%)
Missing	2 (0.0%)	1 (0.0%)	2 (0.0%)	5 (0.0%)
Clinical service				
Did not engage	10,823 (60.7%)	16,141 (73.9%)	18,018 (83.3%)	44,982 (73.4%)
Engaged	7012 (39.3%)	5694 (26.1%)	3608 (16.7%)	16,314 (26.6%)
Completed primary package				
Yes	16,841 (94.4%)	19,712 (90.3%)	18,368 (84.9%)	54,921 (89.6%)
No	994 (5.6%)	2123 (9.7%)	3258 (15.1%)	6375 (10.4%)

### Risks and vulnerabilities

#### By age group

Overall, the most common risks and vulnerabilities among AGYW aged 10–14 years were being a victim of emotional or physical violence or abuse or neglect (78.0%, 95%CI 77.5–78.6) and orphanhood (22.3%, 95%CI 21.7–22.8). Among AGYW aged 15–19 the most common risks and vulnerabilities reported were no/irregular condom use (58.1%, 95%CI 57.5%-58.6%), orphanhood (29.2%, 95%CI 28.7%-29.7%), multiple sexual partners (23.5%, 95%CI 23.0%-24.0%), and transactional sex/sexual exploitation (20.1%, 95%CI 19.7%-20.6%). In the oldest age group, 20–24 years, the most common risks reported included no or irregular condom use (83.5%, 95%CI 82.7%-84.3%), multiple sexual partners (29.9%, 95%CI 28.9%-30.8%), and transactional sex/sexual exploitation (23.0%, 95%CI 22.1%-23.9%).

#### Comparison between age groups

**Fig. 2:**
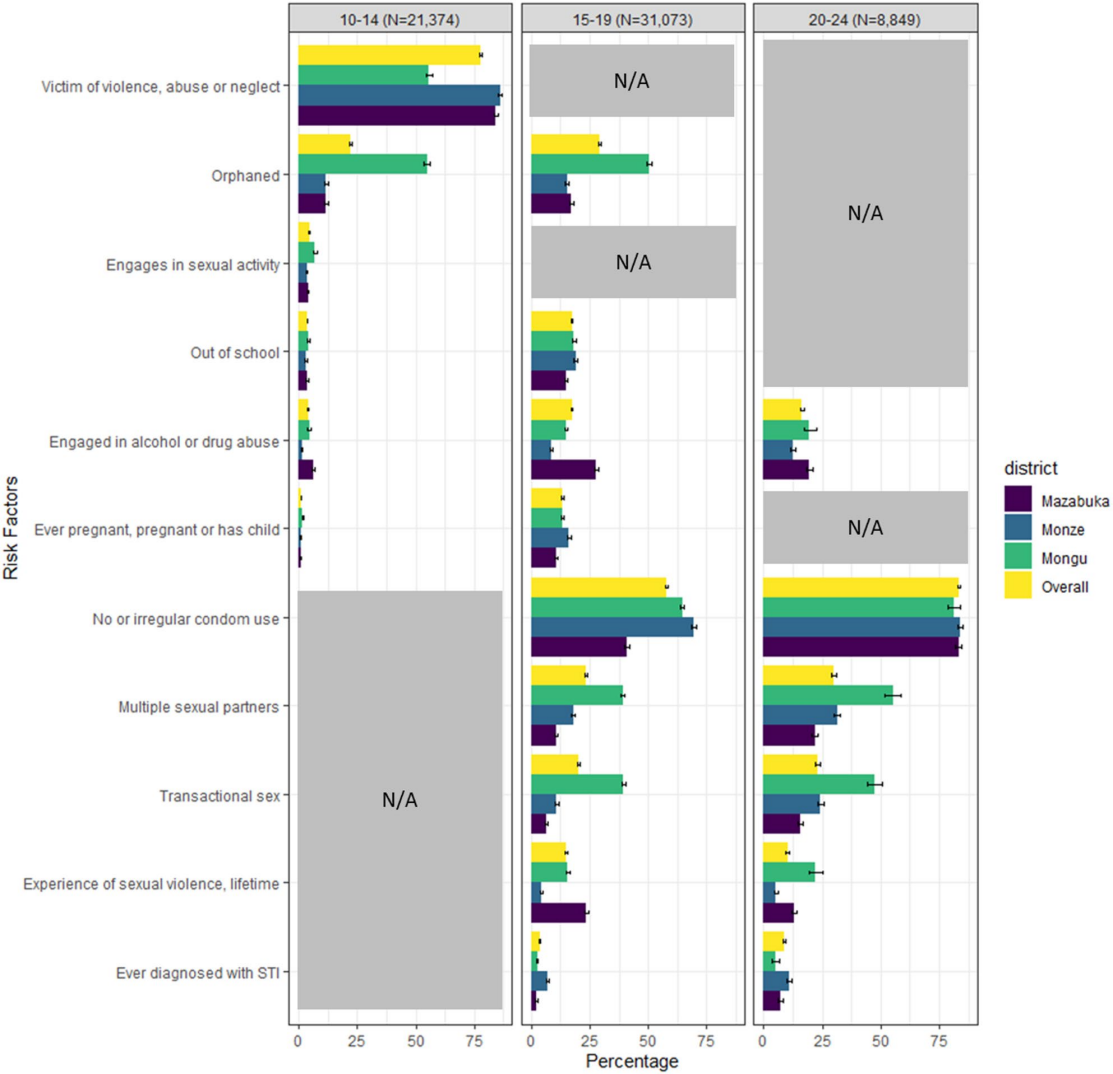
Risks and Vulnerabilities Reported by Age Group (years) and District among DREAMS AGYW, Zambia. October 2020 to August 2023, *N* = 61,296

Drug and alcohol abuse was a risk assessed in all three age groups and reported by 3.9% (95% CI 3.6–4.1%) in the 10–14 years group, 17.8% (95% CI 17.0–17.8%) in the 15–19 years group, and 16.2% (95% CI 15.4–17.0%) in the 20–24 years group. Among the two youngest age groups, 10–14 and 15–19 years, having ever been pregnant or had a child, orphanhood, and being out of school were more frequently reported in the 15–19 years age group (1.0% vs. 13.3, 22.3% vs. 29.2%, and 3.6% vs. 17.4%). Of the risks reported among the two oldest age groups, the proportion reporting multiple sexual partners was higher in the 20–24 years group (23%, 95% CI 23.0–24.0% vs. 29.9%, 95% CI 28.9–30.8%) as was no or irregular condom use (58.1%, 95%CI 57.5%-58.6% vs. 83.5%, 95%CI 82.7–84.3%), and transactional sex/sexual exploitation (20.1%, 95% CI 19.7–20.6% vs. 23.0%, 95% CI 22.1–23.9%) (Fig. 2, Supplemental Table 1).

#### Comparison between districts in Zambia

Across age groups, alcohol or drug abuse was most frequent in Mazabuka, followed by Mongu and Monze (Fig. 2). This was most prominent in the 15–19 years group where 27.8% (95%CI 27.0–28.7%) of AGYW from Mazabuka reported engaging in alcohol or drug abuse. Monze district had the highest proportion of AGYW ever diagnosed with an STI (6.9%, 95% CI 6.4–7.4% in the 15–19 years group and 10.9%, 95%CI 10.0%-11.9% in the 20–24 years group). In the youngest age group, Mazabuka and Monze AGYW reported a higher proportion of being a victim of violence, abuse, or neglect (84.3% in Mazabuka; 86.2% in Monze) compared to Mongu (55.7%). Compared to the two Southern province districts, Mongu AGYW reported the highest proportion of transactional sex/sexual exploitation (39.1%, 95% CI 38.2–40.0% vs. 6% & 11% in the 15–19 years group; and 47.3%, 95% CI 44.0–50.7% vs. 15.6% & 24.3% in the 20–24 years group). Mongu AGYW also reported the highest proportion of sexual activity (7.0% 95%CI 6.3–7.7%) among 10–14-year-olds, orphanhood among 10–14 and 15–19-year-olds (54.8%, 95% CI 53.4–56.1% & 50.4%, 95% CI 49.5–51.3%), and multiple sexual partners among 15-19- and 20-24-year-olds (39.1%, 95% CI 38.2–40.0% & 55.2%, 95% CI 51.9–58.5%).

### Engagement in clinical services

A total of 26.6% (16,314/61,296) of AGYW that were eligible for the DREAMS program engaged in a clinical service. Engagement varied significantly by geographic region with the greatest proportion of AGYW in Mongu (39.3%), followed by Mazabuka (26.1%), and Monze (16.7%). Separate logistic regression models for each age group showing associations between engagement in clinical service and risks and vulnerabilities are summarized in Fig. 2, Supplemental Table 2. Geographic region was significantly associated with engagement in clinical service across all age groups. Compared to Mongu, the Southern districts (Mazabuka and Monze) were significantly less likely to engage in clinical services across all three age groups.

#### By age group

Among AGYW aged 10–14 years, those who were out of school (aOR 2.14, 95% CI 1.47–3.10), engaged in sexual activity (aOR = 1.69, 95% 1.19–2.40), or abused drugs or alcohol (aOR = 1.62, 95% CI 1.11–2.35) were more likely to engage in clinical service compared to those without reporting these risks. Conversely, AGYW who were orphaned (aOR = 0.73, 95% CI 0.56–0.96) and victims of emotional or physical violence or abuse or neglect (aOR = 0.57, 95% CI 0.45–0.73) were less likely to engage in clinical service. Among AGYW aged 15–19 years, those who reported no or irregular condom use (aOR = 1.44, 95% CI 1.36–1.52), had multiple sexual partners in the past year (aOR = 1.22, 95% CI 1.15–1.30), ever been pregnant or pregnant or have a child (aOR = 1.21, 95% CI 1.21–1.31), or were out of school (aOR = 1.16, 95% CI 1.09–1.25) were more likely to engage in clinical service, whereas girls ever diagnosed with STI (aOR = 0.82, 95%C I 0.71–0.94), with experience of sexual violence (aOR = 0.87, 95% CI 0.81–0.93), or engaging in alcohol or drug abuse (aOR = 0.92, 95% CI 0.86–0.98) were less likely to engage in clinical service. Among AGYW aged 20–24 years, those who were ever diagnosed with STI (aOR = 1.20, 95% CI 1.03–1.39) and had multiple sexual partners in the past one year (aOR = 1.16, 1.05–1.28) were more likely to engage in clinical service, whereas girls involved in transactional sex/sexual exploitation (aOR = 0.87, 95% CI 0.78–0.97) were less likely to engage in clinical service.

**Fig. 3:**
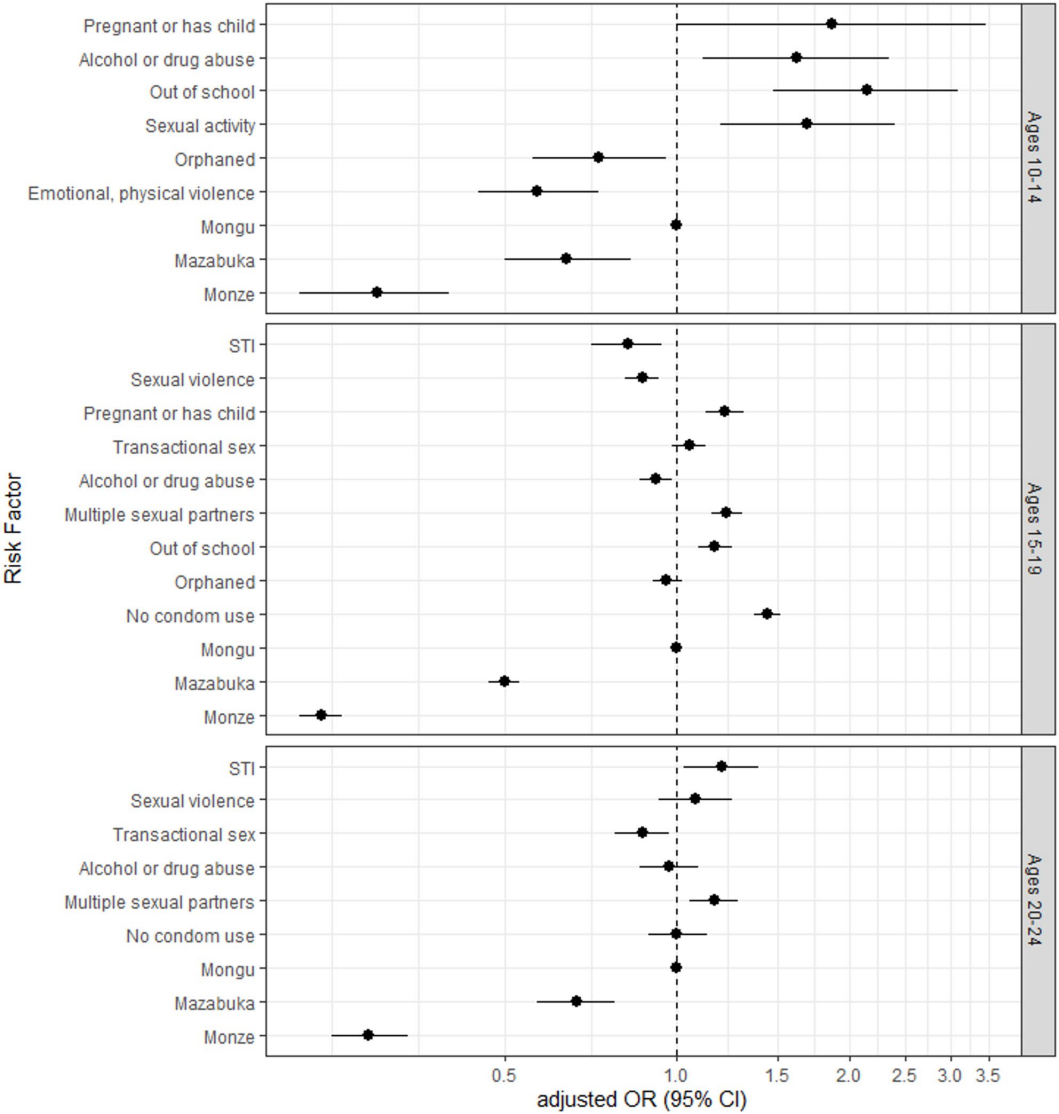
Association between Reported Risks and Clinical Service Engagement among DREAMS AGYW by Age Group (years), Zambia October 2020 to August 2023. Odds ratios presented are the result a multivariable logistic regression model for each age group using binary independent variables except for district where Mongu is the reference category. The dependent variable was engagement in clinical service

In all three models, district was most strongly associated with engagement in clinical service, with engagement being most likely in Mongu (Fig. 3, Supplemental Table 2). In AGYW aged 10–14 years being a victim of emotional or physical violence abuse or neglect and in AGYW aged 15–19 years those who reported sexual violence in their lifetime were both less likely to engage in clinical services compared to those who did not report those risk factors. Being out of school and reporting pregnancy or having a child was associated with clinical service uptake in the 10–14 and 15–19 years AGYW, while reporting multiple sexual partners in the past year was significantly associated in those aged 15–19 and 20–24 years. Those reporting transactional sex/sexual exploitation were significantly less likely to engage in clinical services in the 20–24 years group but there was no association in the 15–19 years group.

## Discussion

In this cross-sectional analysis of HIV risks and vulnerabilities among DREAMS AGYW in Zambia, we identified several prevalent risks by age: GBV and orphanhood among AGYW aged 10–14 years, and low condom use, multiple sexual partners, and transactional sex/sexual exploitation among AGYW aged 15–19 and 20–24 years. While prior studies have assessed common HIV risks among adolescents in Zambia, this represents one of the first characterizations of risks and vulnerabilities among DREAMS AGYW in Zambia. As the DREAMS initiative is no longer being funded by PEPFAR, the positive impact of such targeted approaches for AGYW represent important considerations for Zambian health policy. As the MOH prepares to support AGYW in a post-DREAMS world, understanding the varying risks by age among AGYW can guide future programs to support this vulnerable population.

Our findings on the impact of DREAMS are consistent with prior studies assessing HIV risk factors among AGYW in Zambia. Mathur et al. found that highly vulnerable AGYW in Kenya, Zambia, and Malawi were more likely to have early sexual debut, engage in transactional sex, and experience intimate partner sexual violence [14]. A subsequent study found HIV testing significantly increased among DREAMS AGYW aged 15–19 years, while transactional sex/sexual exploitation increased and sexual violence from non-partners decreased among 20–24-year-olds [15].

### Gender-based violence

We found experience of emotional or physical violence to be highly prevalent (78%) among DREAMS AGYW 10–14 years, particularly in Southern Province (Monze and Mazabuka). This is much higher than reported national estimates of 20.3% and 21% among AGYW under 18 [5, 16], suggesting that DREAMS was appropriately enrolling at-risk girls. This is consistent with other DREAMS studies: in an analysis of risk factors among DREAMS in seven SSA countries, Howard found the most common risk factors among AGYW aged 13-14-years-old was experience of physical/emotional violence, while among AGYW 15-24-year-olds it was lifetime sexual violence [17]. Physical violence tended to decrease with age, while sexual violence increased [17]. Similarly, a Kenyan study found that among DREAMS AGYW aged 10–14 years, over 60% had ever experienced violence, and in the last six months, 33% experienced psychological violence, 16% physical violence, and 5% sexual violence [18].

### Orphanhood

While overall orphanhood in Zambia is 10%, we found that nearly a third (29%) of girls enrolled in DREAMS were orphans, who also had less engagement in clinical services. A multi-country CDC study also found orphanhood to be very high among DREAMS girls, ranging from 18% in Kenya to 44% in Lesotho [17]. This suggests that DREAMS was successfully enrolling at-risk groups such as orphans.

### Low condom use

We identified no or irregular condom use as the most common HIV risk factor reported among older AGYW, which increased with age. This is roughly consistent with national estimates of condom use of 36% and 41% among these age groups at last sexual intercourse [5]. Low condom use was observed among DREAMS girls in other countries as well, ranging from 22% in Zimbabwe to 58% in Uganda [17].

### Multiple sexual partners

In this study, having multiple sexual partners in the last year was reported at over 20% in AGYW 15 years and older; this is much higher than other national estimates of only about 2% [5]. These rates are higher than reported elsewhere for these age categories, with rates of multiple sexual partners among DREAMS girls 15–19 years ranging from 2% in Zimbabwe to 16% in Uganda, and for AGYW 20–24 years from 3% in Zimbabwe to 8.0% in Namibia [17]. This suggests DREAMS accurately identified girls who are sexually active and at risk of HIV.

### Transactional sex

Transactional sex/sexual exploitation among AGYW in this study was a common risk factor and increased with age. Importantly, AGYW aged 20–24 years reporting transactional sex/sexual exploitation were significantly less likely to engage in clinical services. Mathur also found that transactional sex/sexual exploitation increased in this age group, while sexual violence from non-partners decreased [15]. Transactional sex/sexual exploitation was frequently observed among AGYW in SSA, with rates as high as 19% in Uganda [17].

### Clinical services engagement

We examined associations of reported risk factors with engagement in clinical services and found that GBV, orphanhood, and transactional sex/sexual exploitation were negatively associated with engagement in clinical services, while having multiple sexual partners and low condom use were positively associated with clinical services. Multiple sex partners and low condom use were commonly reported among AGYW. GBV and orphanhood likely reflect structural barriers like poverty and low health literacy that hinder healthcare access, while transactional sex/sexual exploitation may be associated with fear of stigma. Higher engagement with clinical services despite these risks suggests growing awareness of HIV prevention and family planning services. However, the lack of condom use among AGYW aged 15–19 years despite clinic engagement indicates possible barriers to accessing condoms or a lack of understanding of their benefits, warranting further investigation.

## Strengths and limitations

This study adds to limited literature on HIV risks and vulnerabilities among DREAMS AGYW in Zambia, an area that is needed to guide further program investment for targeted interventions. We were able to include a large data sample, enabling increased precision of estimates and assessment of associations between risk factors and clinical services.

The primary limitation of this study is the observational nature of our study data. Unmeasured bias may be introduced through confounders and our sample was not randomized. Only girls interested in DREAMS were assessed for risk factors, potentially introducing bias by excluding others in the community. Social desirability may have influenced AGYW to withhold disclosure of certain risk factors. Second, we were not able to control for programmatic factors such as time in program, nor compare DREAMS risk factors to the true prevalence in the communities. Bias could be introduced by measurement error. Third, we found an overwhelmingly large proportion of AGYW screened for eligibility were deemed eligible, potentially due to non-standardized data retention for those who did not report risk. Some risks may be understood differently, resulting in variability among respondents. Additionally, implementing programs receive estimated annual need-based service targets, which may drive service allocation. However, DREAMS staff are trained to offer services to all AGYW who screen as at-risk and eligible, regardless of target achievement. Finally, our program did not assess mental health, which is a significant omission. In South Africa, Mthiyane found that mental health issues were common among AGYW who tested positive for HIV, and also among AGYW who utilized multiple DREAMS interventions pertaining to healthcare [19]. Future DREAMS initiatives may want to consider screening for common mental health issues among AGYW.

## Conclusions

We found high rates of GBV, orphanhood, low condom use, multiple sexual partners, and transactional sex/sexual exploitation in our assessment of HIV risks and vulnerabilities among DREAMS participants in Zambia.

Informed interventions could be developed to respond to AGYW with these risk factors, including government engagement for social protection and welfare (GBV and orphanhood) and innovative programs to improve HIV prevention for AGYW engaged in potentially risky sex. In the emerging post-DREAMS world, these findings may inform more precise programming for AGYW interventions in Zambia and SSA.

## Supplementary Information

Supplementary Material 1.

## Data Availability

Data available upon request.
