# Supplementary material for: HIV risks and vulnerabilities reported among adolescent girls and young women accessing DREAMS services in three Zambian districts: Monze, Mazabuka, and Mongu, 2020–2022
Source: AIDS Res Ther. 2026 Feb 10;23:32. doi: 10.1186/s12981-026-00848-7 (PMC12964886; doi:10.1186/s12981-026-00848-7)
Supplement: Supplementary file 1 — Supplementary Material 1. [file 12981_2026_848_MOESM1_ESM.docx]

**Supplemental Table 1**. Prevalence of Self-Reported risk factors by Age Group and District among DREAMS AGYW, Zambia October 2020 to August 2023, N=61,296

| **Age Group** | **Risk** | **District** | **Yes** | **No** | **Total** | **Percentage** | **Lower CI** | **Upper CI** |
| --- | --- | --- | --- | --- | --- | --- | --- | --- |
| 10-14 years (N=21,374) | Engaged in alcohol or drug abuse | Total | 824 | 20550 | 21374 | 3.9% | 3.6% | 4.1% |
| 10-14 years (N=21,374) | Engaged in alcohol or drug abuse | Mongu | 228 | 4999 | 5227 | 4.4% | 3.8% | 5.0% |
| 10-14 years (N=21,374) | Engaged in alcohol or drug abuse | Mazabuka | 479 | 7248 | 7727 | 6.2% | 5.7% | 6.8% |
| 10-14 years (N=21,374) | Engaged in alcohol or drug abuse | Monze | 117 | 8303 | 8420 | 1.4% | 1.2% | 1.7% |
| 15-19 years (N=31,073) | Engaged in alcohol or drug abuse | Mongu | 1742 | 9962 | 11704 | 14.9% | 14.2% | 15.5% |
| 15-19 years (N=31,073) | Engaged in alcohol or drug abuse | Mazabuka | 2891 | 7496 | 10387 | 27.8% | 27.0% | 28.7% |
| 15-19 years (N=31,073) | Engaged in alcohol or drug abuse | Monze | 766 | 8216 | 8982 | 8.5% | 8.0% | 9.1% |
| 15-19 years (N=31,073) | Engaged in alcohol or drug abuse | Total | 5399 | 25674 | 31073 | 17.4% | 17.0% | 17.8% |
| 20-24 years (N=8,849) | Engaged in alcohol or drug abuse | Mongu | 177 | 727 | 904 | 19.6% | 17.0% | 22.3% |
| 20-24 years (N=8,849) | Engaged in alcohol or drug abuse | Mazabuka | 729 | 2992 | 3721 | 19.6% | 18.3% | 20.9% |
| 20-24 years (N=8,849) | Engaged in alcohol or drug abuse | Monze | 527 | 3697 | 4224 | 12.5% | 11.5% | 13.5% |
| 20-24 years (N=8,849) | Engaged in alcohol or drug abuse | Total | 1433 | 7416 | 8849 | 16.2% | 15.4% | 17.0% |
| 15-19 years (N=31,073) | Ever diagnosed with STI | Mongu | 308 | 11396 | 11704 | 2.6% | 2.3% | 2.9% |
| 15-19 years (N=31,073) | Ever diagnosed with STI | Mazabuka | 225 | 10162 | 10387 | 2.2% | 1.9% | 2.5% |
| 15-19 years (N=31,073) | Ever diagnosed with STI | Monze | 618 | 8364 | 8982 | 6.9% | 6.4% | 7.4% |
| 15-19 years (N=31,073) | Ever diagnosed with STI | Total | 1151 | 29922 | 31073 | 3.7% | 3.5% | 3.9% |
| 20-24 years (N=8,849) | Ever diagnosed with STI | Mongu | 44 | 860 | 904 | 4.9% | 3.6% | 6.5% |
| 20-24 years (N=8,849) | Ever diagnosed with STI | Mazabuka | 265 | 3456 | 3721 | 7.1% | 6.3% | 8.0% |
| 20-24 years (N=8,849) | Ever diagnosed with STI | Monze | 462 | 3762 | 4224 | 10.9% | 10.0% | 11.9% |
| 20-24 years (N=8,849) | Ever diagnosed with STI | Total | 771 | 8078 | 8849 | 8.7% | 8.1% | 9.3% |
| 15-19 years (N=31,073) | Ever pregnant, pregnant or has child | Mongu | 1570 | 10134 | 11704 | 13.4% | 12.8% | 14.0% |
| 15-19 years (N=31,073) | Ever pregnant, pregnant or has child | Mazabuka | 1111 | 9276 | 10387 | 10.7% | 10.1% | 11.3% |
| 15-19 years (N=31,073) | Ever pregnant, pregnant or has child | Monze | 1448 | 7534 | 8982 | 16.1% | 15.4% | 16.9% |
| 15-19 years (N=31,073) | Ever pregnant, pregnant or has child | Total | 4129 | 26944 | 31073 | 13.3% | 12.9% | 13.7% |
| 10-14 years (N=21,374) | Pregnant or have child | Mongu | 85 | 5142 | 5227 | 1.6% | 1.3% | 2.0% |
| 10-14 years (N=21,374) | Pregnant or have child | Mazabuka | 63 | 7664 | 7727 | 0.8% | 0.6% | 1.0% |
| 10-14 years (N=21,374) | Pregnant or have child | Monze | 61 | 8359 | 8420 | 0.7% | 0.6% | 0.9% |
| 10-14 years (N=21,374) | Pregnant or have child | Total | 209 | 21165 | 21374 | 1.0% | 0.9% | 1.1% |
| 15-19 years (N=31,073) | Experience of sexual violence, lifetime | Mongu | 1833 | 9871 | 11704 | 15.7% | 15.0% | 16.3% |
| 15-19 years (N=31,073) | Experience of sexual violence, lifetime | Mazabuka | 2442 | 7945 | 10387 | 23.5% | 22.7% | 24.3% |
| 15-19 years (N=31,073) | Experience of sexual violence, lifetime | Monze | 375 | 8607 | 8982 | 4.2% | 3.8% | 4.6% |
| 15-19 years (N=31,073) | Experience of sexual violence, lifetime | Total | 4650 | 26423 | 31073 | 15.0% | 14.6% | 15.4% |
| 20-24 years (N=8,849) | Experience of sexual violence, lifetime | Mongu | 200 | 704 | 904 | 22.1% | 19.5% | 25.0% |
| 20-24 years (N=8,849) | Experience of sexual violence, lifetime | Mazabuka | 478 | 3243 | 3721 | 12.8% | 11.8% | 14.0% |
| 20-24 years (N=8,849) | Experience of sexual violence, lifetime | Monze | 221 | 4003 | 4224 | 5.2% | 4.6% | 5.9% |
| 20-24 years (N=8,849) | Experience of sexual violence, lifetime | Total | 899 | 7950 | 8849 | 10.2% | 9.5% | 10.8% |
| 15-19 years (N=31,073) | No or irregular condom use | Mongu | 7548 | 4156 | 11704 | 64.5% | 63.6% | 65.4% |
| 15-19 years (N=31,073) | No or irregular condom use | Mazabuka | 4253 | 6134 | 10387 | 40.9% | 40.0% | 41.9% |
| 15-19 years (N=31,073) | No or irregular condom use | Monze | 6250 | 2732 | 8982 | 69.6% | 68.6% | 70.5% |
| 15-19 years (N=31,073) | No or irregular condom use | Total | 18051 | 13022 | 31073 | 58.1% | 57.5% | 58.6% |
| 20-24 years (N=8,849) | No or irregular condom use | Mongu | 737 | 167 | 904 | 81.5% | 78.8% | 84.0% |
| 20-24 years (N=8,849) | No or irregular condom use | Mazabuka | 3099 | 622 | 3721 | 83.3% | 82.0% | 84.5% |
| 20-24 years (N=8,849) | No or irregular condom use | Monze | 3554 | 670 | 4224 | 84.1% | 83.0% | 85.2% |
| 20-24 years (N=8,849) | No or irregular condom use | Total | 7390 | 1459 | 8849 | 83.5% | 82.7% | 84.3% |
| 10-14 years (N=21,374) | Out of school | Mongu | 215 | 5012 | 5227 | 4.1% | 3.6% | 4.7% |
| 10-14 years (N=21,374) | Out of school | Mazabuka | 283 | 7444 | 7727 | 3.7% | 3.3% | 4.1% |
| 10-14 years (N=21,374) | Out of school | Monze | 265 | 8155 | 8420 | 3.1% | 2.8% | 3.5% |
| 10-14 years (N=21,374) | Out of school | Total | 763 | 20611 | 21374 | 3.6% | 3.3% | 3.8% |
| 15-19 years (N=31,073) | Out of school | Mongu | 2141 | 9563 | 11704 | 18.3% | 17.6% | 19.0% |
| 15-19 years (N=31,073) | Out of school | Mazabuka | 1554 | 8833 | 10387 | 15.0% | 14.3% | 15.7% |
| 15-19 years (N=31,073) | Out of school | Monze | 1699 | 7283 | 8982 | 18.9% | 18.1% | 19.7% |
| 15-19 years (N=31,073) | Out of school | Total | 5394 | 25679 | 31073 | 17.4% | 16.9% | 17.8% |
| 10-14 years (N=21,374) | Engages in sexual activity | Mongu | 364 | 4863 | 5227 | 7.0% | 6.3% | 7.7% |
| 10-14 years (N=21,374) | Engages in sexual activity | Mazabuka | 303 | 7424 | 7727 | 3.9% | 3.5% | 4.4% |
| 10-14 years (N=21,374) | Engages in sexual activity | Monze | 284 | 8136 | 8420 | 3.4% | 3.0% | 3.8% |
| 10-14 years (N=21,374) | Engages in sexual activity | Total | 951 | 20423 | 21374 | 4.4% | 4.2% | 4.7% |
| 15-19 years (N=31,073) | Transactional sex | Mongu | 4615 | 7089 | 11704 | 39.4% | 38.5% | 40.3% |
| 15-19 years (N=31,073) | Transactional sex | Mazabuka | 657 | 9730 | 10387 | 6.3% | 5.9% | 6.8% |
| 15-19 years (N=31,073) | Transactional sex | Monze | 980 | 8002 | 8982 | 10.9% | 10.3% | 11.6% |
| 15-19 years (N=31,073) | Transactional sex | Total | 6252 | 24821 | 31073 | 20.1% | 19.7% | 20.6% |
| 20-24 years (N=8,849) | Transactional sex | Mongu | 428 | 476 | 904 | 47.3% | 44.0% | 50.7% |
| 20-24 years (N=8,849) | Transactional sex | Mazabuka | 581 | 3140 | 3721 | 15.6% | 14.5% | 16.8% |
| 20-24 years (N=8,849) | Transactional sex | Monze | 1025 | 3199 | 4224 | 24.3% | 23.0% | 25.6% |
| 20-24 years (N=8,849) | Transactional sex | Total | 2034 | 6815 | 8849 | 23.0% | 22.1% | 23.9% |
| 15-19 years (N=31,073) | Multiple sexual partners | Mongu | 4579 | 7125 | 11704 | 39.1% | 38.2% | 40.0% |
| 15-19 years (N=31,073) | Multiple sexual partners | Mazabuka | 1098 | 9289 | 10387 | 10.6% | 10.0% | 11.2% |
| 15-19 years (N=31,073) | Multiple sexual partners | Monze | 1617 | 7365 | 8982 | 18.0% | 17.2% | 18.8% |
| 15-19 years (N=31,073) | Multiple sexual partners | Total | 7294 | 23779 | 31073 | 23.5% | 23.0% | 23.9% |
| 20-24 years (N=8,849) | Multiple sexual partners | Mongu | 499 | 405 | 904 | 55.2% | 51.9% | 58.5% |
| 20-24 years (N=8,849) | Multiple sexual partners | Mazabuka | 816 | 2905 | 3721 | 21.9% | 20.6% | 23.3% |
| 20-24 years (N=8,849) | Multiple sexual partners | Monze | 1328 | 2896 | 4224 | 31.4% | 30.0% | 32.9% |
| 20-24 years (N=8,849) | Multiple sexual partners | Total | 2643 | 6206 | 8849 | 29.9% | 28.9% | 30.8% |
| 10-14 years (N=21,374) | Orphaned | Mongu | 2863 | 2364 | 5227 | 54.8% | 53.4% | 56.1% |
| 10-14 years (N=21,374) | Orphaned | Mazabuka | 909 | 6818 | 7727 | 11.8% | 11.1% | 12.5% |
| 10-14 years (N=21,374) | Orphaned | Monze | 990 | 7430 | 8420 | 11.8% | 11.1% | 12.5% |
| 10-14 years (N=21,374) | Orphaned | Total | 4762 | 16612 | 21374 | 22.3% | 21.7% | 22.8% |
| 15-19 years (N=31,073) | Orphaned | Mongu | 5895 | 5809 | 11704 | 50.4% | 49.5% | 51.3% |
| 15-19 years (N=31,073) | Orphaned | Mazabuka | 1801 | 8586 | 10387 | 17.3% | 16.6% | 18.1% |
| 15-19 years (N=31,073) | Orphaned | Monze | 1365 | 7617 | 8982 | 15.2% | 14.5% | 16.0% |
| 15-19 years (N=31,073) | Orphaned | Total | 9061 | 22012 | 31073 | 29.2% | 28.7% | 29.7% |
| 10-14 years (N=21,374) | Victim of violence, abuse, or neglect | Mongu | 2911 | 2316 | 5227 | 55.7% | 54.3% | 57.0% |
| 10-14 years (N=21,374) | Victim of violence, abuse, or neglect | Mazabuka | 6511 | 1216 | 7727 | 84.3% | 83.4% | 85.1% |
| 10-14 years (N=21,374) | Victim of violence, abuse, or neglect | Monze | 7256 | 1164 | 8420 | 86.2% | 85.4% | 86.9% |
| 10-14 years (N=21,374) | Victim of violence, abuse, or neglect | Total | 16678 | 4696 | 21374 | 78.0% | 77.5% | 78.6% |

**Supplemental Table 2**. Association between Reported Risks and Clinical Service Engagement among DREAMS AGYW by Age Group, Zambia October 2020 to August 2023, N=61,296

|  | **Did not engage in Clinical Service** | **Engaged in Clinical Service** | **uOR** | **P-value** | **aOR** | **P-value** |
| --- | --- | --- | --- | --- | --- | --- |
| **Eligibility Criteria/Risks Reported** | **(N=44,982)** | **(N=16,314)** |  |  |  |  |
| **Ages 10-14 years** | **(N=20,998)** | **(N=376)** |  |  |  |  |
| Pregnant or have a child |  |  |  |  |  |  |
| No | 20,801 (98.3%) | 197 (94.3%) | ref |  | ref |  |
| Yes | 364 (1.7%) | 12 (5.7%) | 3.48 (1.93-6.29) | <0.001 | 1.86 (1.0-3.46) | 0.05 |
| Abuses drugs or alcohol |  |  |  |  |  |  |
| No | 20,210 (98.3%) | 788 (95.6%) | ref |  | - ref |  |
| Yes | 340 (1.7%) | 36 (4.4%) | 2.71 (1.91-3.86) | <0.001 | **1.62 (1.11-2.35)** | 0.011 |
| Out of school |  |  |  |  |  |  |
| No | 20,270 (98.3%) | 728 (95.4%) | ref |  | Ref |  |
| Yes | 341 (1.7%) | 35 (4.6%) | 2.86 (2.0-4.08) | <0.001 | **2.14 (1.47-3.10)** | <0.001 |
| Engages in sexual activity |  |  |  |  |  |  |
| No | 20,088 (98.4%) | 910 (95.7%) | ref |  | Ref |  |
| Yes | 335 (1.6%) | 41 (4.3%) | 2.70 (1.94-3.76) | <0.001 | **1.69 (1.19-2.40)** | 0.003 |
| Orphaned |  |  |  |  |  |  |
| No | 16,343 (98.4%) | 4,655 (97.7%) | ref |  | Ref |  |
| Yes | 269 (1.6%) | 107 (2.3%) | 1.40 (1.11-1.75) | 0.004 | **0.73 (0.56-0.96)** | 0.027 |
| Victim of emotional or physical violence or abuse or neglect |  |  |  |  |  |  |
| No | 4,548 (96.9%) | 16,450 (98.6%) | ref |  | Ref |  |
| Yes | 148 (3.1%) | 228 (1.4%) | 0.43 (0.34-0.52) | <0.001 | **0.57 (0.45-0.73)** | <0.001 |
| District |  |  |  |  |  |  |
| Mongu | 5,067 (96.9%) | 160 (3.1%) | ref |  | ref |  |
| Mazabuka | 7,581 (98.1%) | 146 (1.9%) | 0.61 (0.49-0.76) | <0.001 | **0.64 (0.50-0.83)** | 0.001 |
| Monze | 8,350 (99.2%) | 70 (0.8%) | 0.26 (0.20-0.35) | <0.001 | **0.30 (0.22-0.40)** | <0.001 |
|  |  |  |  |  |  |  |
| **Ages 15-19 years** | **(N=19,510)** | **(N=11,563)** |  |  |  |  |
| Ever diagnosed with STI |  |  |  |  |  |  |
| No | 18,707 (62.5%) | 803 (69.8%) | ref |  | ref |  |
| Yes | 11,215 (37.5%) | 348 (30.2%) | 0.72 (0.64-0.82) | <0.001 | **0.82 (0.71-0.94)** | 0.004 |
| Experience of sexual violence, lifetime |  |  |  |  |  |  |
| No | 16,555 (62.6%) | 2,955 (63.5%) | ref |  | ref |  |
| Yes | 9,868 (37.4%) | 1,695 (36.5%) | 0.96 (0.90-1.03) | 0.245 | **0.87 (0.81-0.93)** | <0.001 |
| Ever been pregnant or pregnant or has a child |  |  |  |  |  |  |
| No | 17,188 (63.8%) | 2,322 (56.2%) | ref |  | ref |  |
| Yes | 9,756 (36.2%) | 1,807 (43.8%) | 1.37 (1.28-1.46) | <0.001 | **1.21 (1.12-1.31)** | <0.001 |
| Transactional sex including staying in a relationship for material or financial support |  |  |  |  |  |  |
| No | 16,360 (65.9%) | 3,150 (50.4%) | ref |  | ref |  |
| Yes | 8,461 (34.1%) | 3,102 (49.6%) | 1.90 (1.80-2.01) | <0.001 | 1.05 (0.98-1.12) | 0.175 |
| Engaged in alcohol or drug abuse |  |  |  |  |  |  |
| No | 16,047 (62.5%) | 3,463 (64.1%) | ref |  | ref |  |
| Yes | 9,627 (37.5%) | 1,936 (35.9%) | 0.93 (0.88-0.99) | 0.024 | **0.92 (0.86-0.98)** | 0.013 |
| Had multiple sexual partners in the past one year |  |  |  |  |  |  |
| No | 15,729 (66.1%) | 3,781 (51.8%) | ref |  | ref |  |
| Yes | 8,050 (33.9%) | 3,513 (48.2%) | 1.81 (1.72-1.91) | <0.001 | **1.22 (1.15-1.30)** | <0.001 |
| Out of school |  |  |  |  |  |  |
| No | 16,410 (63.9%) | 3,100 (57.5%) | ref |  | ref |  |
| Yes | 9,269 (36.1%) | 2,294 (42.5%) | 1.31 (1.23-1.39) | <0.001 | **1.16 (1.09-1.25)** | <0.001 |
| Orphaned |  |  |  |  |  |  |
| No | 14,412 (65.5%) | 5,098 (56.3%) | ref |  | ref |  |
| Yes | 7,600 (34.5%) | 3,963 (43.7%) | 1.47 (1.40-1.55) | <0.001 | 0.96 (0.91-1.02) | 0.211 |
| No or irregular condom use |  |  |  |  |  |  |
| No | 8,926 (68.5%) | 10,584 (58.6%) | ref |  | ref |  |
| Yes | 4,096 (31.5%) | 7,467 (41.4%) | 1.54 (1.47-1.61) | <0.001 | **1.44 (1.36-1.52)** | <0.001 |
| District |  |  |  |  |  |  |
| Mongu | 5,465 (46.7.9%) | 6,239 (53.3%) | ref |  | ref |  |
| Mazabuka | 7,007 (67.5%) | 3,380 (32.5%) | 0.42 (0.40-0.45) | <0.001 | **0.50 (0.47 -0.53)** | <0.001 |
| Monze | 7,038 (78.4.2%) | 1,944 (21.6%) | 0.24 (0.23-0.26) | <0.001 | **0.24 (0.22-0.26)** | <0.001 |
|  |  |  |  |  |  |  |
| **Ages 20-24 years** | **(N=4,474)** | **(N=4,375)** |  |  |  |  |
| Ever diagnosed with STI |  |  |  |  |  |  |
| No | 4,093 (50.7%) | 381 (49.4%) | ref |  | ref |  |
| Yes | 3,985 (49.3%) | 390 (50.6%) | 1.05 (0.91-1.22) | 0.506 | **1.20 (1.03-1.39)** | 0.021 |
| Experience of sexual violence, lifetime |  |  |  |  |  |  |
| No | 4,084 (51.4%) | 390 (43.4%) | ref |  | ref |  |
| Yes | 3,866 (48.6%) | 509 (56.6%) | 1.38 (1.20-1.58) | <0.001 | 1.08 (0.93-1.25) | 0.321 |
| Engaged in alcohol or drug abuse |  |  |  |  |  |  |
| No | 3,781 (51.0%) | 693 (48.4%) | ref |  | ref |  |
| Yes | 3,635 (49.0%) | 740 (51.6%) | 1.11 (0.99-1.24) | 0.069 | 0.97 (0.86-1.09) | 0.577 |
| Transactional sex including staying in a relationship for material or financial support |  |  |  |  |  |  |
| No | 3,423 (50.2%) | 1,051 (51.7%) | ref |  | ref |  |
| Yes | 3,392 (49.8%) | 983 (48.3%) | 0.94 (0.85-1.04) | 0.253 | **0.87 (0.78-0.97)** | 0.012 |
| Had multiple sexual partners in the past one year |  |  |  |  |  |  |
| No | 3,201 (51.6%) | 1,273 (48.2%) | ref |  | ref |  |
| Yes | 3,005 (48.4%) | 1,370 (51.8%) | 1.15 (1.05-1.26) | 0.003 | **1.16 (1.05-1.28)** | 0.003 |
| No or irregular condom use |  |  |  |  |  |  |
| No | 728 (49.9%) | 3,746 (50.7%) | ref |  | ref |  |
| Yes | 731 (50.1%) | 3,644 (49.3%) | 0.97 (0.86-1.08) | 0.580 | 1.00 (0.89-1.13) | 0.962 |
| District |  |  |  |  |  |  |
| Mongu | 291 (32.2%) | 613 (67.8%) | ref |  | ref |  |
| Mazabuka | 1,553 (41.7%) | 2,168 (58.3%) | 0.66 (0.57-0.77) | <0.001 | **0.67 (0.57-0.78)** | <0.001 |
| Monze | 2,630 (62.3%) | 1,594 (37.7%) | 0.29 (0.25-0.33) | <0.001 | **0.29 (0.25-0.34)** | <0.001 |

***Supplemental l***

**Determined Resilient Empowered AIDS-free Mentored Safe (DREAMS) Initiative**

**DREAMS Screening Form**

| **Implementing Partner** | |  | |  | **Province** |  | |  | |  | | **Identifier’s name** | |  | | |
| --- | --- | --- | --- | --- | --- | --- | --- | --- | --- | --- | --- | --- | --- | --- | --- | --- |
| **District** | |  | |  | **Site Name** |  | |  | |  | | **Identifier’s Phone #** | |  | | |
| **AGYW’s surname** | |  | |  | **Phone #** |  | |  | |  | | **Date of Screening** | | **DD / MM / YYYY** | | |
| **AGYW’s middle name** | |  | |  |  |  | |  | |  | |  | |  | | |
| **AGYW’s first name** | |  | |  | **NRC #** |  | | **/** | | **/** | | **Name of Guardian** | |  | | |
| **AGYW’s Date of birth** | | **DD / MM / YYYY** | | | **Physical address** |  | |  | |  | | **Cell phone of Guardian** | |  | |  |
| **AGYW’s birth order** | |  | | | **AGYW’s province of birth** |  | |  | |  | | **Sex (indicate 01)** | |  | |  |
|  | |  | |  |  |  | |  | |  | |  | |  | | |
| **10-14 years** | |  | |  |  |  | |  | |  | |  | |  | | |
| AGYW meeting at **least one** of the following criteria will be eligible for enrolment on DREAMS | | | | | | | | | |  | |  | | *Tick all that apply* | |  |
| Q1: Ever been pregnant or pregnant or has a child | | | | | | | |  | |  | |  | |  | |  |
| Q2: Victim of emotional or physical violence or abuse or neglect | | | | | | | | | | | | | |  | |  |
| Q3: Alcohol or drug use | | | | | | | | | | | | | |  | |  |
| Q4: Out of school | |  | |  |  |  | |  | |  | |  | |  | | |
| Q5: Orphan hood | | | |  |  |  | |  | |  | |  | |  | | |
| Q6: Engages in sexual activity | | | |  |  |  | |  | |  | |  | |  | | |
| **Comments** | |  | |  |  |  | |  | |  | |  | |  | | |
|  | |  | |  |  |  | |  | |  | |  | |  | | |
| **Eligible** (Yes or No) | |  | |  |  |  | |  | |  | |  | |  | | |
|  | |  | |  |  |  | |  | |  | |  | |  | | |
| **15-19 years** | |  | |  |  |  | |  | |  | |  | |  | | |
| AGYW meeting at **least one** of the following criteria will be eligible for enrolment on DREAMS | | | | | | | | | |  | |  | | *Tick all that apply* | |  |
| Q1: Had multiple sexual partners in the past one year | | | | | | | |  | |  | |  | |  | |  |
| Q2: Ever been pregnant or pregnant or has a child | | | | | | | |  | |  | |  | |  | |  |
| Q3: Have you ever been diagnosed with an STI | | | | | | | |  | |  | |  | |  | |  |
| Q4: No or Irregular condom use | | | |  |  |  | |  | |  | |  | |  | | |
| Q5: Transactional sex including staying in a relationship for material or financial support | | | | | | | |  | |  | |  | |  | |  |
| Q6: Experience of sexual violence (Lifetime) | | | | | | | | | |  | |  | |  | |  |
| Q7: Is engaged in alcohol or drug abuse | | | | | | | | | |  | |  | |  | |  |
| Q8: Out of school | | | | | | | |  | |  | |  | |  | |  |
| Q9: Orphan hood | |  | |  |  |  | |  | |  | |  | |  | | |
| **Comments** | |  | |  |  |  | |  | |  | |  | |  | | |
| **Eligible** (Yes or No) | |  | |  |  |  | |  | |  | |  | |  | | |
|  | |  | |  |  |  | |  | |  | |  | |  | | |
| **20-24 years** | | | |  |  |  | |  | |  | |  | |  | | |
| AGYW meeting at **least one** of the following criteria will be eligible for enrolment on DREAMS | | | | | | | | | | | |  | | *Tick all that apply* | |  |
| Q1: Had multiple sexual partners in the past one year | | | | | | | | | | | | | |  | |  |
| Q2: Have you ever been diagnosed with an STI | | | | | | | |  | |  | |  | |  | |  |
| Q3: No or Irregular condom use | | | |  |  |  | |  | |  | |  | |  | | |
| Q4: Transactional sex including staying in a relationship for material or financial support | | | | | | | |  | |  | |  | |  | |  |
| Q5: Experience of sexual violence (Lifetime) | | | | | | | | | |  | |  | |  | |  |
| Q6: Is engaged in alcohol or drug abuse | | | |  |  |  | |  | |  | |  | |  | | |
| **Comments** | |  | |  |  |  | |  | |  | |  | |  | | |
| **Eligible** (Yes or No) | |  | |  |  |  | |  | |  | |  | |  | | |
|  | |  | |  |  |  | |  | |  | |  | |  | | |
| **Enroll in DREAMS (Yes/No)** | |  | |  |  |  | |  | |  | |  | |  | | |
| **Screening Location**  **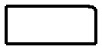** | |  | |  |  |  | |  | |  | |  | |  | | |
| School | | Clinic | | 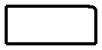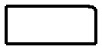 Community | | | |  | |  | |  | |  | |  |
